# Comparative Study of Methods for Cycle Length Estimation in Fractionated Electrograms of Atrial Fibrillation

**DOI:** 10.3390/jpm12101712

**Published:** 2022-10-13

**Authors:** Diego Osorio, Aikaterini Vraka, José Moreno-Arribas, Vicente Bertomeu-González, Raúl Alcaraz, José J. Rieta

**Affiliations:** 1 BioMIT.org, Electronic Engineering Department, Universitat Politecnica de Valencia, 46022 Valencia, Spain; 2 Cardiology Department, Saint John’s University Hospital, 03550 Alicante, Spain; 3 Research Group in Electronic, Biomedical and Telecommunication Engineering, University of Castilla-La Mancha, 16071 Cuenca, Spain

**Keywords:** atrial fibrillation, electrogram, complex fractionated atrial electrograms, local activation waves, detection, invasive recordings, cycle length, comparison

## Abstract

Atrial cycle length (CL) is an important feature for the analysis of electrogram (EGM) characteristics acquired during catheter ablation (CA) of atrial fibrillation (AF), the commonest cardiac arrhythmia. Nevertheless, a robust ACL estimator requires the precise detection of local activation waves (LAWs), which still remains a challenge. This work aims to compare the performance in (CL) estimation, especially under fractionated EGMs, of three different LAW detection methods relying on different operation strategies. The methods are based on the hyperbolic tangent (HT) function, an adaptive amplitude threshold (AAT) and a (CL) iteration (ACLI), respectively. For each method, LAW detection has been assessed with respect to manual annotations made by two experts and performance has been estimated by confusion matrix and mean and individual (CL) error calculation by EGM types of fractionation. The influence of EGM length on the individual (CL) error has been additionally considered. For the HT method, accuracy, sensitivity and precision were 92.77–100%, while for the AAT and ACLI methods they were 78.89–99.91% for all EGM types. The CL error on the HT method was lower than AAT and ACLI methods (up to 12 ms versus up to 20 ms), with the difference being more prominent in complex EGMs. The HT method also showed the lowest dependency on EGM length, presenting the lowest and least variable error values. Therefore, the HT method achieves higher performance in (CL) estimation in comparison with previous LAW detection techniques. The high robustness and precision demonstrated by this method suggest its implementation on CA mapping devices for a more successful location of ablation targets and improved results during CA procedures.

## 1. Introduction

Being the most common cardiac arrhythmia in clinical practice, atrial fibrillation (AF) affects more than 43 million people worldwide [1]. The risks of stroke, myocardial infarction, heart failure or dementia are increased in patients suffering from AF, which is also associated with increased mortality [1,2]. The ever-growing life expectancy in comparison to the high AF prevalence for older individuals [1] and the significant economic burden that AF provokes [1,3] highlight the need for an efficient treatment.

So far, catheter ablation (CA) is considered the star AF treatment due to its high effectiveness and safety [1,4,5]. The aim of the CA procedure is to eliminate the ectopic atrial beats that can trigger or sustain AF by electrically isolating the areas that are thought to present arrhythmogenic activity or fibrosis, respectively [1,6]. Pulmonary veins (PVs) are the most common AF foci, especially for patients in paroxysmal AF [1,7,8]. Nevertheless, other atrial sites may contribute to AF perpetuation, especially in persistent AF patients, where the atrial remodeling takes place to a higher extent [1,9]. Consequently, additional CA may be necessary in order to achieve freedom from AF [10]. Areas targeted for additional CA are defined via electrogram (EGM) analysis, which allows the detection of remodeled tissue favoring AF perpetuation or even triggering AF. Low-voltage, high dominant frequency (DF), low AF cycle length (CL) or highly complex EGMs, called complex fractionated atrial electrograms (CFAEs), are some of the most established techniques to define candidate CA targets, albeit showing controversial results [11,12,13,14,15,16]. These types of EGMs are thought to correspond to areas of slow conduction or pivotal points of reentrant wavelets, revealing the presence of atrial fibrosis which provokes AF perpetuation [17].

For many of the aforementioned techniques, the detection of local activation waves (LAWs) is an elemental step. In low fractionated EGMs, LAWs can be easily detected, hence facilitating the analysis [18]. However, in CFAEs and highly fractionated EGMs, LAWs detection can become a hard task often misleading the CL estimation and, consequently, the procedures and assessments based on this feature [13]. Moreover, the possibility of ambiguous LAWs annotations in CFAEs may be an explanation for the low impact of non-PV CA on the freedom from AF [14,15]. Therefore, the development and disposal of a reliable and robust LAW estimator are of paramount importance. Although various approaches to the development of LAWs detectors have been attempted so far [19,20,21,22,23,24,25,26,27], most of them focus on unipolar EGMs [20,21,25,26,27], where LAWs detection is a much easier task [13]. Nevertheless, as LAWs detection in bipolar EGMs is more complicated due to the nonstationary nature of the recorded dynamics as well as the high dependency on the wavefront direction, not but a few techniques for bipolar EGMs have been developed so far [19,22,23,24].

One of the first and most cited works employed an adaptive amplitude-based threshold which varied according to the last detected peaks, showing low error values for regular or low fractionated EGMs but increased rates for fractionated signals [19]. The CL-based method is another highlighted work [22]. Search is performed with an initial amplitude threshold which is then modified according to CL. This method has also shown low error rates for regular EGMs. Nevertheless, when CL becomes irregular, as normally happens in CFAEs, error rates increase. The DF-based method detects LAWs corresponding to periodic peaks of the EGMs [23]. Although this method showed good performance compared to previous methods regarding periodic EGMs, it also failed to show satisfactory results for aperiodic signals, which is a much more frequent event in highly fractionated EGMs. Thus, it will not be considered in this study. Finally, the newer of the aforementioned methods is based on the application of the hyperbolic tangent (HT) function on CFAEs in order to highlight and facilitate low amplitude detections as well as to limit very high amplitude activations using a hybrid methodology combining attributes of the CL- and adaptive amplitude-methods, showing high performance in CFAEs and complex EGMs [24].

Given the advantage of bipolar over unipolar EGMs in being insensitive to ventricular activity and the high use of the former in AF studies, the appropriate strategy choice for works that may need to implement LAWs detection in their analysis can be quite confusing yet inevitable. Despite the results shown in the aforementioned studies, each one of them has used a different database. Hence, a direct comparison would be unfair or would not be directly performed. The main objective of the present work is to perform a straightforward comparison between the three most robust aforementioned LAW detectors, the adaptive amplitude threshold (AAT) [19], the atrial CL iterator (ACLI) method [22] and the hyperbolic tangent (HT) method [24], by reproducing each one of them and comparing the results over the same database. Since the common denominator of LAWs detection in any of the aforementioned studies is the CL estimation, using this parameter as a performance evaluation metric seems a fair option. Comparison is performed over a range of factors that could affect or create biased results, such as the length of EGMs or different fractionation levels. In this way, a multidimensional assessment can be performed in order to offer a more reliable and direct comparative measure for future studies.

The manuscript is organized as follows. Section 2 presents the dataset used in this study and the preprocessing methods applied, as well as a brief presentation of the methodology of the strategies under comparison and the evaluation methods used. Section 3 includes the results of the comparison, while Section 4 provides an overview of the methods under comparison and analyzes the main conclusions derived from the results, which are then summarized in Section 5.

## 2. Methods

The dataset employed in this study consisted of 119 EGMs of 10 s in length, recorded by a Labsystem™ PRO EP recording system (Boston Scientific, Marlborough, MA, USA) after obtaining the written consent of 22 patients undergoing CA procedures. EGMs were sampled at 1 kHz and filtered by an adaptive notch filter to reduce powerline interference together with a 0.5–500 Hz band-pass filter to limit high-frequency noise. The dataset was manually annotated and classified by EGM types by two expert physicians according to Wells’ classification [28]. Type I EGMs consisted of clear LAWs and almost isoelectric baseline, type II contained low perturbations in the baseline and a higher number of deflections at each LAW while type III contained a high fluctuating baseline and low-amplitude fragmented LAWs. Sixteen EGMs were classified by the experts as AF type I, 19 as AF type II and 84 as AF type III, so that the vast majority of the EGMs selected pertained to the most difficult AF type to delineate. Signals were denoised by a Wavelet Transform-based technique, which has been proved to perform better than regular filtering, providing quick results, effective noise elimination and optimal preservation of the signal morphology [29].

### 2.1. LAW Detection Methods

The methodology of each of the studied LAW detection strategies is presented briefly in the next subsections, followed by a comparative example of the preprocessing method adopted by each strategy.

#### 2.1.1. Adaptive Amplitude Threshold Method

The first method implemented was the AAT algorithm [19]. This technique is based on Botteron and Smith preprocessing [18], a technique developed to generate waveforms proportional to the amplitude of the EGM components with frequencies within the band-pass filter cutoffs. This method includes 40–250 Hz band-pass filtering, followed by signal rectification and low-pass filtering with the cut-off frequency at 20 Hz. The AAT algorithm employs an AAT based on the last ten detected activations with exponentially decreasing weights ($s_{w}$), resulting from the application of the Botteron filtering, using a blanking period of 55 ms between activations. Lastly, the modulus of the original signal is filtered by a non-causal moving average filter with 90 coefficients, resulting in a new signal $s_{f}$, where the positive zero crossings closer to a local peak of $s_{w}$ define the activation times. A more detailed description of the algorithm can be found elsewhere [19].

#### 2.1.2. Atrial Cycle Length Iteration Method

The ACLI method [22] is based on a modified Botteron and Smith [18] preprocessing, replacing the band-pass filter with a 40 Hz high-pass filter and increasing the low-pass filter cut-off to 30 Hz. The algorithm performs an iterative LAW detection, starting with the highest amplitude peak and moving to the next peak according to an amplitude descending order, making use of a blanking period of 50 ms, until the average CL is lower than 275 ms. Once this condition is fulfilled, a second condition has to be accomplished so that the iteration stops: either the mean CL has to be less than the median CL plus 5 ms or the amplitude of the current peak has to be 20% less than the amplitude of the previous peak. Finally, a loop control checks the existence of intervals longer than 1.5 × the median CL and adds, in case such intervals are found, the highest peaks within these intervals to the activation set. More details on the methodology can be found elsewhere [22].

#### 2.1.3. Hyperbolic Tangent Method

The HT method [24] also modifies the Botteron and Smith preprocessing steps, expanding the range of the bandpass filter cutoffs from 40–250 to 20–250 Hz. Studies on the original preprocessing effect on CFAEs demonstrated that slower components of activations were being lost, provoking an insufficient waveform response, leading the algorithm to miss the respective activations [24]. Therefore, a reduction in the minimum band-pass filter cut-off was necessary, with 20 Hz being the optimal frequency based on analyses performed.

The preprocessing of this method also used a novel technique, the HT function, aiming to reduce high amplitude variability observed in CFAEs in order to facilitate the detection process that follows next. For this purpose, each recording is segmented into 7-second intervals with 25% overlapping and classified according to their fractionation level by their kurtosis calculated at each 1 s and averaged, so that the HT function is applied exclusively on CFAEs. The detection process is initiated by an amplitude-based localization of all peaks higher than 0.4 mV, taking into consideration the minimum refractory period of 50 ms. The process continues with the median CL calculation and a secondary search on intervals longer than the median CL is performed, reducing the amplitude threshold proportionally to the difference between the median CL and the current interval. The process finishes with the calculation of the barycenter of each LAW, defined as the sample point that equally divides the area of the modulus of the LAWs, in order to estimate the activation time. More details on this algorithm are provided elsewhere [24].

**Figure 1:**
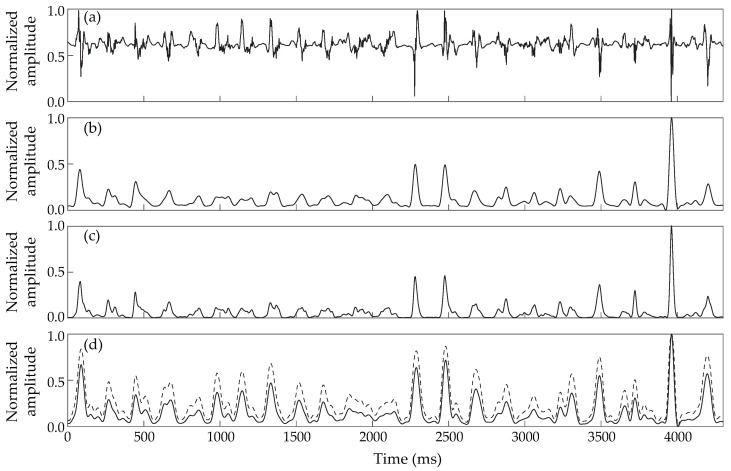
Comparative analysis of the preprocessing stage of each of the compared LAW detection methods applied on the same EGM: (**a**) Denoised EGM to be processed; (**b**) Result of AAT preprocessing; (**c**) Result of ACLI preprocessing; (**d**) Result of HT preprocessing. The dashed line in (**d**) corresponds to the application of the hyperbolic tangent.

**Figure 2:**
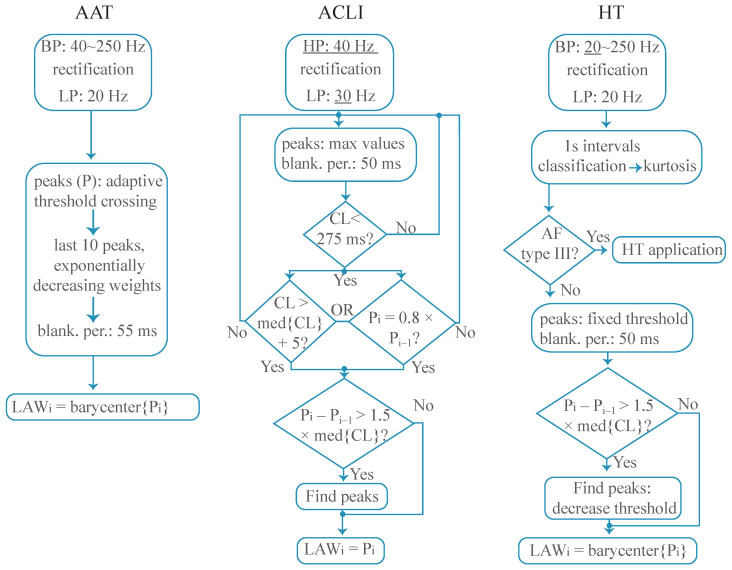
Block diagrams of the LAW detection methods compared. Underlined values in the first blocks show modified thresholds with respect to traditional Botteron’s preprocessing method. BP: band-pass filtering; LP: low-pass filtering; HP: high-pass filtering; $P_{i}$: amplitude of the *i*-th peak; med{CL}: median cycle length.

A comparative example of these three different preprocessing strategies applied over the same EGM is shown in Figure 1, where it can be observed how the HT method produces a signal in which activations are easier to detect automatically in later stages because small peaks are enhanced and large peaks are limited. Additionally, block diagrams of each technique’s methodology are presented in Figure 2.

### 2.2. Performance Evaluation

Confusion matrix and CL error metrics were employed in order to evaluate the performance. Regarding the confusion matrix, *Accuracy*, *Sensitivity* and *Precision* were calculated. True positive (TP) were the LAWs annotated both by the experts and by the method under analysis. False positives (FP) were the LAWs that were only annotated by the method, while false negatives (FN) were the LAWs that were annotated only by the experts. Since there are no true negatives (TN), these metrics were calculated as follows:

(1) $$Accuracy=\frac{TP}{TP+FP+FN},$$

(2) $$Sensitivity=\frac{TP}{TP+FN},$$

and

(3) $$Precision=\frac{TP}{TP+FP}.$$

Details on CL error calculation are provided next. The effect of EGMs duration as well as fractionation has also been considered.

#### 2.2.1. Detection Performance

The number of LAWs successfully detected at each method was calculated by comparing the annotations made by the algorithm with the manual annotations made by the experts, allowing a threshold of 40 ms. Performance was evaluated in terms of Accuracy, Sensitivity and Precision, providing an overall perspective on performance, including the undersensing and oversensing rates of each algorithm.

#### 2.2.2. Mean CL Error Estimation

**Figure 3:**
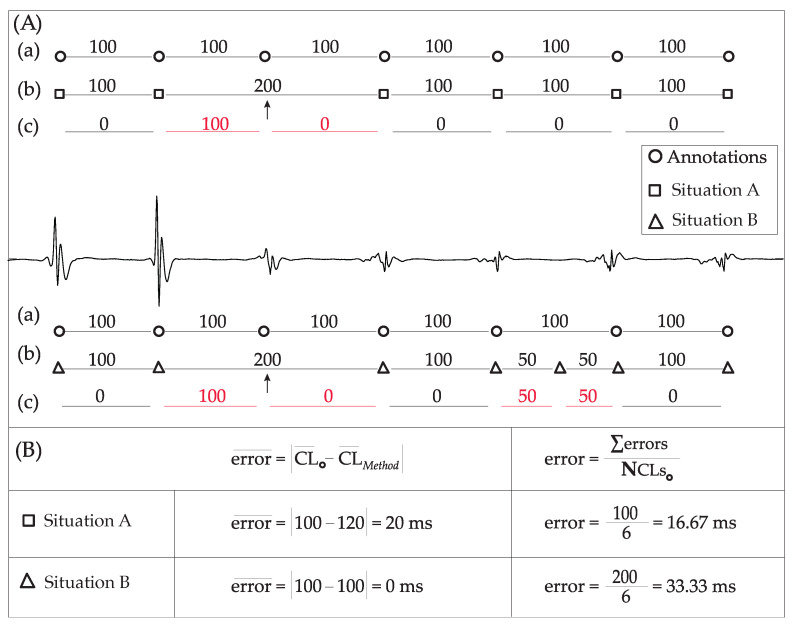
Comparative example for calculating the CL estimation error. (**A**) EGM with experts’ (circle) and method’s annotations: (a) expert’s CL (b) method’s CL and (c) errors in CL estimation. Top: Situation 1 (squares) undersensing case (missing activation-red). The arrow shows the starting points in case of unequal CLs for the undersensing case; Bottom: Situation 2 (triangles) undersensing and oversensing case (false positive activation-red). (**B**) Error estimation for the two example situations. (left) Mean CL error; (right) Individual CL error. Notice how the simultaneous existence of under- and oversensing in Situation 2 may create erroneous CL calculation for the mean CL case.

The error in estimating the average CL at any of the three methods was measured as the absolute difference between the mean CL of the manual and algorithm’s annotations, as can be seen from Figure 3, which will be explained more in-depth later for individual CL error estimation. Nevertheless, considering that oversensing or undersensing can have an effect on the mean CL, producing misleading results as can be seen in Figure 3B, mean CL error can not be considered as a reliable performance indicator. Thus, an analysis of individual CL errors was additionally developed in order to obtain more realistic results.

#### 2.2.3. Individual CL Error Estimation

As previously stated, this metric was introduced in order to reliably calculate the CL error for each algorithm, without any bias inserted from possible under- or oversensing tendencies. In this case, the error at each CL is defined by the absolute difference between the manual annotations, called reference annotations, and the algorithm’s annotations. The individual CL error is finally calculated by the sum of errors found at each CL divided by the number of reference intervals. The formula for the calculation of the individual CL error is given in the right column of Figure 3B. If the CL was the same for the manual and algorithm annotations, the respective error was set to zero. However, if there was some under- or oversensing detected, the evaluation process was able to quantify the error.

For the undersensing case, as the given CL was longer for the algorithm than for the manual annotations case, the corresponding error is calculated by subtracting the reference CL from the algorithm’s CL. However, in that case, the next interval under comparison will involve the following reference segment, while the algorithm’s segment will remain the same as before, considered to be starting from the reference segment’s initiation point so that a double counting on error will be avoided. An example of this process can be seen in Figure 3A, with experts’ (circle) and method’s (square, triangle) annotations. The top panel shows Situation 1 (square) with an undersensing case where the arrow shows the beginning of the algorithm’s segment (missed activation-red). For each interval between annotations, line (a) indicates the expert’s CL, line (b) the method’s CL and line (c) errors in the estimation of each CL. The bottom panel shows Situation 2 (triangles) with both undersensing (missed activation-red) and oversensing cases (falsely detected activation-red). Regarding the oversensing case, the error at the given interval will be calculated by subtracting the algorithm’s CL from the reference CL, for each one of the algorithm’s intervals between a reference interval, as can be seen from Situation 2 of Figure 3A. Finally, as the mean CL of Situation 1, with one missed activation, is 120 ms, then the mean error is 20 ms. Nevertheless, the simultaneous existence of under- and oversensing in Situation 2 will create an erroneous mean CL calculation of 0 ms, as this method had a mean CL of 100 ms, even though not all the activations corresponded to the experts’ annotations. On the contrary, the individual error of Situation 2 was 33.33 ms, both indicating a non-perfect annotator and showing a higher error than Situation 1 (16.67 ms), which only had one mishit.

#### 2.2.4. Influence of Electrograms Duration

Another aspect analyzed in this study was the influence of the EGMs duration on the individual CL error of each method. For each type of EGM, the three methods were applied progressively from 1 to 10 s of duration over the dataset, computing the individual CL error as a measure of the CL estimation precision.

## 3. Results

### 3.1. Detection Results

**Figure 4:**
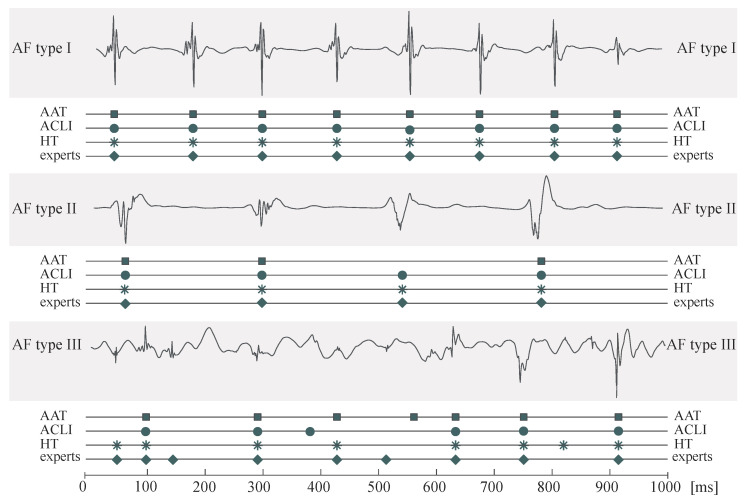
One-second example of the three EGM types and the annotations made from the three algorithms as well as from the experts. Experts’ annotations are considered as ground truth. For AF type I, activations were clearly distinguished from the baseline and all the methods were able to identify them. For AF type II, although the baseline shows little perturbation, lower amplitude activations slightly complicate the procedure, leading to a mishit for the AAT algorithm. Finally, for AF type III EGMs, mishits and false positives are observed for all methods across the EGM.

An example of the three AF-type EGMs along with LAWs annotations from experts and the compared techniques can be seen in Figure 4. While LAWs detection is pretty straightforward for AF types I and II, LAWs detection in AF type III EGMs is significantly complicated, with each algorithm presenting some false positive or negative detections. Results on detection performance for the methods under comparison are shown in Table 1 for EGMs of all AF types. Both ACLI and HT methods show optimal results for types I and II. On the contrary, the AAT method showed lower accuracy and precision rates, which is especially unusual for AF type I. This fact is provoked by the high variability in amplitude observed in several type I EGMs of the dataset used for the present analysis. Figure 3 corresponds to a type I EGM example, in which the impact of amplitude variability on the AAT method’s detection performance can be seen. Although AAT’s accuracy rate is not lower than 90% in type I EGMs, this relatively low performance implies significant deficiencies of the AAT method and a higher CL error, provoked by the longer CL of AF type I EGMs, leading to the lower number of intervals and, consequently, the higher weight of each mishit.

The results for type III EGMs, also shown in Table 1, are of great importance in understanding the performance of each method, as this type of EGM coincides with the most fragmented signals and CFAEs. Firstly, the AAT method shows the lowest accuracy results, while sensitivity and precision are quite compensated, with values of 85.38% and 91.11%, respectively, due to higher undersensing than oversensing incidents. The ACLI method shows improved results in comparison with the AAT method. However, a quite intense discrepancy can be seen between undersensing and oversensing tendencies of this method, as can be observed from the difference between sensitivity and precision rates at 87.51% and 96.89%, respectively. Finally, the HT method presents the most satisfactory results for all confusion matrix parameters, with a balance observed between undersensing and oversensing tendencies, expressed by the 95.30% and 97.24% of sensitivity and precision values, respectively, while showing the highest accuracy rates as well.

### 3.2. Cycle Length Estimation

**Figure 5:**
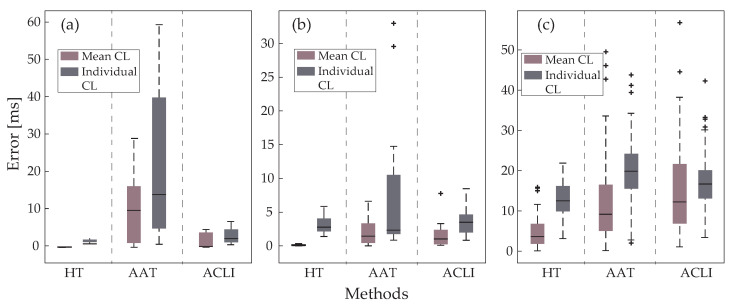
Comparative analysis of errors estimating CL using the mean CL and the individual CL methods for the three LAW detectors compared: (**a**) Results for type I electrograms; (**b**) type II electrograms; and (**c**) type III electrograms. Values indicated with + stand for outliers.

Regarding CL results, Figure 5a shows the mean and individual CL errors on type I EGMs. The mean CL error in ACLI and HT methods is close to 0, as expected from the high detection accuracy and the equilibrium between sensitivity and precision. Individual CL error in these methods is also quite low, yet slightly higher than mean CL error since bias from undersensing or oversensing behavior is removed in this parameter. Regarding AAT results, both mean and individual CL error are significantly higher, at about 10 ms and 12 ms, respectively. This is explained by the relatively low accuracy of this method in type I EGMs. As before, higher individual CL errors are explained by the balance between the undersensing and oversensing rates of these EGM types.

The results for type II EGMs are presented in Figure 5b. ACLI and HT methods show results similar to those from type I EGMs, with an increased difference between individual and mean CL errors. As previously mentioned, mean CL estimation for the AAT method compensated for the errors provoked by under- or oversensing. Lower amplitude variability in the employed dataset led to better performance in AF type II than type I EGMs for the AAT method, as was observed from the detection results. According to this performance improvement, the error in type II EGMs was lower than type I for the AAT method.

Finally, Figure 5c shows the CL error on type III EGMs, which include CFAEs. Being the most complicated case, analysis of this type of EGM clearly demonstrates that individual CL error estimation is a more suitable parameter, efficiently removing any impact of external factors on the CL error calculation, as can be seen from the discrepancies between the mean and individual CL error estimation of each method, leading to invalid conclusions in the case of the AAT and ACLI methods. Error on all methods is higher in this EGM type, with the HT technique showing the best results both in mean (about 4 ms in HT vs. 9 ms in AAT vs. 11 ms in ACLI) and individual (about 12 ms in HT vs. 20 ms in AAT vs. 17 ms in ACLI) CL error analysis.

### 3.3. EGM Length Influence

**Figure 6:**
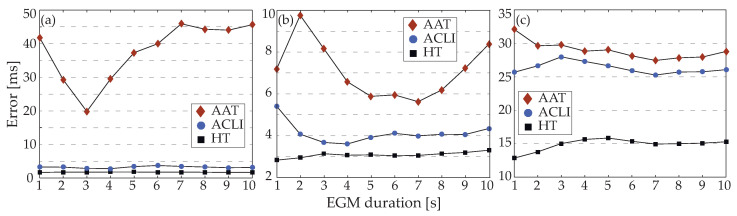
Individual CL error depending on EGM length for the three LAW detection methods analyzed: (**a**) Results for type I electrograms; (**b**) type II electrograms; and (**c**) type III electrograms.

The results of the application of each method over the dataset for different EGM lengths are presented in Figure 6, where individual CL error is analyzed. The HT method shows the most stable results regarding length, being additionally improved in comparison with the other methods. For type I EGMs, the ACLI method shows similar results. As the fractionation level increases, however, ACLI performance falls, showing significantly worse results. Finally, AAT error is the highest regarding all AF types, while this method also shows a higher dependency on the EGM length.

## 4. Discussion

Due to the high importance of precise AF substrate mapping, several methods for LAW detection have been developed [19,20,22,23,24,25,26] until the present. However, some of these techniques cannot be reproduced or directly applied on CA devices, due to either the particularity of the database [20] used or to the controversial methodology followed, using computational models premised on assumptions about tissue’s geometry [25,26]. Other techniques can be directly implemented on substrate mapping devices, such as the AAT [19], the ACLI [22], the dominant frequency (DF) [23] and the HT [24] methods. Each one of these studies has shown promising results, the correct choice for future works in need of a reliable LAWs detector, though, can be confusing as the database used is different.

The present study has performed a direct comparison of three of the aforementioned methods, the AAT, the ACLI and the HT method. The reason why the DF method was not included in the comparison is the lack of individual CL estimation of the DF method, which is one of the main evaluation parameters of the present study. In addition, the DF method failed to provide satisfactory results under aperiodic signals, which is the most typical situation in highly fractionated EGMs.The results indicate that HT is the most robust and reliable method, showing higher accuracy and precision and lower CL errors, suggesting its recruitment for both future studies and CA devices.

The high performance of the HT method can be assigned to the alternative preprocessing strategy and the recruitment of the HT function, which prevented the detector from missing low amplitude LAWs in CFAEs. What is more, this method also adopted a segmentation strategy that allowed the adaptation to various signal lengths, while the combination of an adaptive amplitude threshold with CL correction allowed the detection of LAWs in segments with high amplitude variability [24]. Finally, activation time estimation through the barycenters led to an estimation closer to the experts’ annotation.

### 4.1. Evaluation on Different Levels of Fractionation

EGM fractionation plays a significant role in the performance of each method. Normally, in low fractionation environments, performance is high and only a few errors are observed. Thus, low performance in low fractionation EGMs implies a lack of reliability. As EGMs become more fractionated, with indiscernible activations, the LAW detector’s accuracy tends to drop. However, since CFAEs, which are highly fractionated EGMs, are the main challenge of any LAW detector, the performance of each method on that kind of recording is crucial and serves as an index to evaluate reliability and robustness.

This study has utilized the Wells’ classification for types of EGMs, with ascending levels of fractionation from type I to type III EGMs. In this way, insufficient results in type I EGMs indicate a less reliable method, while competent results on type III EGMs affirm the reliability of another method. The final assessment is a trade-off among the performance of each EGM type, with special weight to type III EGMs, which are the distinguishing parameter.

The HT method showed the highest results in terms of a confusion matrix for all EGM types, being considered this way the most reliable method, achieving high performance. On the contrary, the AAT method showed relatively low results in AF type I EGMs, due to the high amplitude variability present in this EGM type of the current database.

### 4.2. Cycle Length as an Evaluation Parameter

The main implementation of LAWs detection in real-time mapping devices is related to CL estimation. Consequently, the error in CL estimation was included in the evaluation procedure. As it was shown, mean CL error can be affected by under- or oversensing incidence, and hence, its employment is not recommended. Contrarily, individual CL estimation is an impartial method. Individual CL error was the lowest in the HT method for all EGMs, implying again the high reliability and performance of this technique, while the AAT and ACLI methods have shown poor results, especially for type III EGMs.

### 4.3. Effect of Signal Length

Adaptability and proper function regardless of external factors are keys to optimized performance. As signal length varies from study to study, the ability of an algorithm to perform optimally under any length should be taken into consideration for the evaluation of its performance. Additionally, signal length could affect the conclusions of the conducted comparison. For the aforementioned reasons, the individual CL error on each method was assessed over various EGM lengths.

The robustness of the HT algorithm was proved not only by the fact that the error was minimal for any EGM type and length but also because it was not significantly affected by the signal length. This means that this method can perform regardless of the duration of the database used, providing flexibility both to prospective studies and to CA devices. On the other side, the AAT method showed the highest individual error for all EGM types, especially high for EGM type I. Moreover, this algorithm showed unusually high dependence on EGM length for types I and II EGMs, possibly due to high discrepancies between amplitude values in various segments of the same recording.

## 5. Conclusions

The HT method is an optimal LAW detector, showing higher performance than the AAT and ACLI techniques in every aspect under investigation. The high accuracy and robustness of the HT method contribute to precise CL length estimation, regardless of the signal length or the EGM fractionation. The high adaptability of this method suggests its implementation on real-time mapping devices aiming to localize CA targets or its adoption by future studies, providing reliable LAW detection results and precise CL estimation.

## Figures and Tables

**Table 1:** Electrogram detection performance results for all AF types of the three LAW detectors compared in this study in terms of Accuracy (Acc), Sensitivity (Se) and Precision (Pr).

Method	Type I			Type II			Type III		
	Acc [%]	Se [%]	Pr [%]	Acc [%]	Se [%]	Pr [%]	Acc [%]	Se [%]	Pr [%]
AAT	91.82	95.60	95.66	95.90	97.35	98.33	78.89	85.38	91.11
ACLI	98.58	99.91	98.68	98.78	99.73	99.05	85.13	87.51	96.89
HT	100.00	100.00	100.00	100.00	100.00	100.00	92.77	95.30	97.24

## Data Availability

The data supporting reported results and presented in this study are available on request from the corresponding author.
